# Compulsory School In- and Outdoors—Implications for School Children’s Physical Activity and Health during One Academic Year

**DOI:** 10.3390/ijerph13070699

**Published:** 2016-07-12

**Authors:** Peter Pagels, Anders Raustorp, Peter Guban, Andreas Fröberg, Cecilia Boldemann

**Affiliations:** 1 Department of Sport Sciences, Linnaeus University, SE-391 82 Kalmar, Sweden; anders.raustorp@gu.se; 2 Department of Public Health Sciences, Karolinska Institutet, SE-171 77 Stockholm, Sweden; cecilia.boldemann@ki.se; 3 Department of Food and Nutrition and Sport Science, University of Gothenburg, SE-405 30 Gothenburg, Sweden; andreas.froberg@gu.se; 4 Center for Epidemiology and Community Medicine, Stockholm County Council, Box 1497, 171 29 Solna, Sweden; peter.guban@sll.se

**Keywords:** health, children, school environment, physical activity, physical education

## Abstract

Regulated school days entail less free-living physical activity (PA) and outdoor stay, which may jeopardize the opportunities for cohesive moderate-to-vigorous physical activity (MVPA) and, by extension, children’s health. The role of outdoor stay during school time for pupils’ free-living PA vs. physical education (PE) and indoor stay was studied during one academic year in 196 pupils aged 7–14 years at four schools in mid-southern Sweden during five consecutive days each in September, March, and May. Actigraph GT3X+ Activity monitors were used. Predictors for PA during school stay were expressed as mean daily accelerometer counts and were measured per season, day, grade, gender, weather, and time outdoors. Overall, free-living PA outdoors generated the highest mean accelerometer counts for moderate and vigorous PA. Outdoor PA and PE, representing 23.7% of the total school time contributed to 50.4% of total mean accelerometer counts, and were the greatest contributors to moderate and vigorous PA. Age and weather impacted PA, with less PA in inclement weather and among older pupils. More time outdoors, at all seasons, would favorably increase school children’s chances of reaching recommended levels of PA.

## 1. Introduction

Between ages 6–15, children spend most of their waking hours during workdays at school, making the school environment an important factor in maintaining and promoting health (amongst others) by offering sufficient amounts of daily physical activity (PA) [1,2,3]—particularly outdoors where it is additionally beneficial for motor control and vitamin D formation [4]. PA during school time has also been reported to improve students’ health and academic outcomes, particularly among low-income and minority children [5]. Free-living outdoor activity, such as running, climbing, and scampering, contributes to verbal, emotional, and social development [6]. It is further a mighty contributor to consolidating active lifestyles in childhood and puberty, with a positive impact on health and hence reduced risk of common diseases in adulthood [7,8,9]. The transition from preschool to long regulated school days may jeopardize the opportunities for cohesive moderate-to-vigorous physical activity (MVPA) due to less free-living PA and less outdoor stay, thereby increasing the risk of developing unhealthy conditions that increasingly manifest themselves before puberty [10,11].

Consensus guidelines urge children and adolescents to perform ≥60 min of moderate-to-vigorous PA (MVPA) on most days of the week to promote health benefits to be sustained into adulthood [9,12,13]. Depending on the cut-off point for accelerometer counts per minute (CPM) applied to define MVPA, at best about 87% of children and adolescents accumulate recommended levels of PA when using the most cited cut-off point of >2000 CPM [14]. However, with a cut-off point of >3000 CPM, only 3% obtain these recommended levels [14].

In cross-sectional studies, PA levels are reported to decline by age [15,16,17], which is also confirmed in a longitudinal Swedish study [18]. Season is also relevant in analyzing behaviors surrounding PA [19]. Some studies have observed declining PA during winter in 8–11 year old children [16,20,21]. Evidence also concludes that girls are generally less physically active than boys during school recess [15,22,23], but it is not known if this applies to the whole school day. Only few previous objectively measured studies of school children’s PA include measurements during an entire school week [1] several times during one academic year [13]. Additionally, no known study has applied methods of high validity in separating outdoor from indoor PA, and likewise in separating PA generated by scheduled physical education (PE) from that generated by free-living PA. As children move before and between classes, as well as during classes (depending on the subject of the lesson, etc*.*), PA outside recess is also relevant. School day segments such as scheduled recess and PE have been identified as an area to endorse pupils’ PA. The World Health Organization (WHO) recommends that PE should be considered as an integrated part of creating health-promoting environments [24]. At school, free-living PA most frequently occurs outdoors during recess [16] and evidence shows that pupils accumulate more MVPA during recess outdoors than indoors in gyms or classrooms [25]. Indoor activity during school time is reported to be mainly characterized by light PA (LPA) and lacking cohesive MVPA activity of at least five minutes [26].

To explore the contribution of various scenarios to PA—and ultimately the chances for pupils to obtain recommended levels of PA—the role of pupils’ free-living PA and PE in- and outdoors during school time was studied during one academic year.

## 2. Methods

### 2.1. Research Design and Participants

A repeated measurement study design was used, investigating differences between indoor and outdoor school time, and scheduled PE vs. free-living PA during school time upon pupils’ PA during fall, late winter, and late spring. The study was approved by the Regional Ethics Committee of Stockholm, Item# 2011/370-31.

The sample was drawn from second (integrated age classes 7–10 years old), fifth (11–13 years old), and eighth graders (14–15 years old) at four municipal schools in mid-southern Sweden. The schools—each attended by 400–500 first to ninth graders—were selected to reflect the socio-economic composition of the municipalities of the whole country, with a majority of the population living in medium-sized cities outside or on the outskirts of a metropolitan area (Statistics Sweden). The ISCO code was applied for socioeconomic classification along the European socio-economic classification, ISCO, 1988.

The parents of 259 children received relevant and detailed information and were given the opportunity to discuss the study before signing a written consent to let their children participate, and 196 (76%) pupils (second graders = 78, fifth graders = 89, and eighth graders = 29) agreed to participate.

### 2.2. Procedures

Data collection was carried out during one to five consecutive school days on three occasions during the school year, fall (September 2012), late winter (March 2013), and late spring (May 2013). The pupils were weighed (scale: GS 27 Happystripes (Beurer, Utrecht, The Netherlands)) and waists and heights measured using a measuring tape and a stadiometer (Seca 217 (Seca, Birmingham, UK)). Body mass index (BMI) was calculated (kg/m^2^). For confounder control, bivariate analysis was carried out from diary records (bedtime, wake-up time, medication, feeling well/unwell, PA outside school), none of them being significantly related to MVPA.

The weather conditions were observed and ranked AM and PM each day of fieldwork according to the following weather index: 1 = cloudless, 2 = partly cloudy, 3 = white cloudiness, 4 = grey cloudiness, 5 = precipitation. Temperature recordings were obtained from the Swedish Metrological and Hydrological Institute (SMHI).

### 2.3. Physical Activity Assessments

PA was measured with accelerometers (Actigraph GT3X+ Activity monitors (Actigtaph, Pensacola, FL, USA)), which enable time-stamped analysis of duration, intensity, and location of activity in terms of indoor or outdoor activity, due to built-in light sensors (Actilux (Actigtaph)) with a sensitivity of 74% and specificity of 86% [27], and valid for separating outdoor time in free-living children [28]. This supplemented the separation of outdoor from indoor time recorded by ocular observation. The accelerometers were activated on the first day and analyzed upon each data collection period—i.e., after five consecutive days of three study periods.

The accelerometers were set to record movement in ten second epochs with a sampling-fervency of 60 Hz to get detailed PA data [29,30]. In 2011 Trost et al. made a comparison of accelerometer cut points to predict activity intensity in 5–15-year-old participants, and the cut-off point of >2296 CPM for MVPA by Evenson et al. [31] was shown to provide best classification accuracy among children of all ages and is thus recommended as optimal for estimating children’s and adolescents’ time spent in MVPA [31,32]. Thus, the outcome was summarized into 1-min data and analyzed with the recommended cut-points for vertical axis data set to ≤100 CPM for sedentary (SED), 101–2295 CPM for LPA, 2296–4011 CPM for moderate PA (MPA), and ≥4012 for vigorous PA (VPA) [31,32].

Ambient light data were sampled at a rate of 1 Hz and downloaded with epoch lengths of 10 s, showing average lux values for each epoch. The cut point for the lux value explaining outdoor time was set to >130 lux, after comparing with ocular observation for in- and outdoor data, which were fairly consistent with newly presented validations of the GT3X+ light sensor [27,28].

At each school, one experienced observer was in charge of one class each, tracking the pupils’ indoor and outdoor time during the whole school day. Accelerometers were put on upon arrival and removed at departure from school.

### 2.4. Statistical Analysis

SAS for Windows software packages (version 9.4, SAS Institutet Inc., Cary, NC, USA) were used for analysis. Accelerometer data were processed using Acti Life 6.0 (Actigtaph) and Microsoft Excel 2010 (Microsoft Corporation, Redmond, WA, USA). Alpha level was set at 0.05 (*p* ≤ 0.05). The mean and standard deviations (±SD) for total accelerometer counts (total counts), CPM, and daily accelerometer counts attained in four PA levels (SED, LPA, MPA, and VPA) in- and outdoors during free-living PA and PE were calculated. Differences between individual students’ average in- and outdoor CPM and MVPA, both free-living and PE, were tested with Wilcoxon Sign Rank test for all participants and within genders.

For each season, individual accelerometer data from at least two days and ≥120 min including ≥5 min outdoors per day per participant were included for analysis. A *t*-test was performed, comparing the group qualifying for inclusion criteria for analysis of accelerometry data vs. the group that did not, considering MVPA indoors (a few pupils were not outdoors at all), and no significant difference between the two groups was observed. The mean numbers of measured school days per child were 3.9 (±0.9), 4.3 (±0.9), and 4.1 (±0.9) in September, March, and May, respectively. To evaluate how PA was affected by outdoor school time, gender, weather, grades, weekdays, and seasons during stay at the school premises, a generalized linear model using generalized estimating equation with repeated measurements was applied in the GENMOD procedure. The models had total daily accelerometer counts, CPM, and MVPA counts as dependent variable in their respective model with time spent outdoors, temperature, gender, grade, weekday, season, and weather type as independent variables. All independent variables were included in all models to achieve mutual adjustment. For confounder control, bivariate analysis was carried out from diary records (medication, feeling well/unwell, PA outside school), none of them being significantly related to MVPA. Socioeconomic status was not related to physical activity as previously shown in the study group [16].

## 3. Results

Sufficient available data from 179 participants (92 boys and 87 girls) with a total of 1854 valid measured activity days were analyzed. Table 1 shows descriptive data of the participants and time outdoors during different seasons.

### 3.1. Time Indoors vs. Outdoors

Overall, 76.3% of the entire time at the school premises was spent indoors. Indoor stay dominated during all seasons, with a mean of 267.8 (±66.2) min indoors and 83.9 (±55.5) min outdoors (time outdoors + time indoors = school day). Mean outdoor stays differed between seasons and were 87.2 (±55.5) min in September, 66.0 (±50.6) min in March, and 98.1 (±55.3) min in May. The second graders spent significantly more time outdoors during the school day across all three seasons, with a mean of 110.0 (±57.0) min, the fifth graders 71.0 (±47.2) min, and eighth graders 36.9 (±34.4) min (*p* < 0.001). The mean duration of outdoor PE classes was 51 min in September and 46 min in May. No outdoor PE was scheduled in March. Table 1 shows time spent outdoors in different grades and seasons.

### 3.2. Predictors for Physical Activity during School Stay

Predictors for PA during school stay were season, measured day, grade, gender, weather, and time outdoors, which all significantly impacted physical activity. Being a girl, indoor stay, being a fifth or eighth grader, and grey cloudiness were predictors for decreased PA. The strongest predictor for increased PA was being a boy, time outdoors, being a second grader, the first and the second day of the week, and weather (clear skies) (Table 2).

### 3.3. Physical Activity Outdoors vs. Indoors

**Figure 1:**
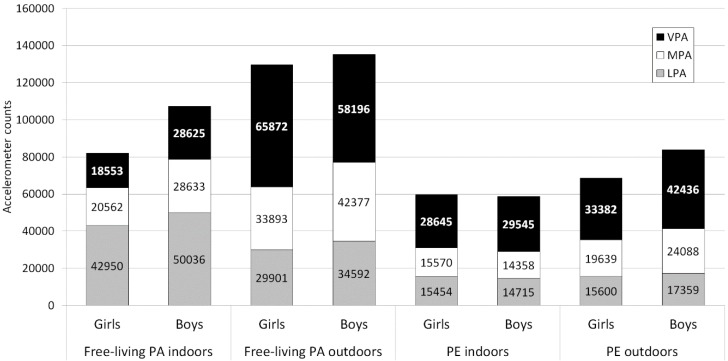
Mean daily total accelerometer counts (total staple) divided in different physical activity (PA) levels indoors and outdoors during time spent in free-living PA or physical education (PE), Sweden 2012–2013; LPA: Light PA, MPA: Moderate PA, VPA: Vigorous PA.

Overall, outdoor free-living PA generated the highest mean accelerometer counts for both MPA and VPA, which applied to both genders (Figure 1). In total, outdoor PA (free-living PA + PE) generated in average 50.4% (±24.3) of the total accelerometer counts (139,189 ± 122,531), corresponding to 23.7% of school time during which these counts were generated. Conversely, indoor PA generated in average of 44.1% (±24.7) of the total accelerometer counts (100,664 ± 73,294) during 76.3% of the time spent at school.

**Figure 2:**
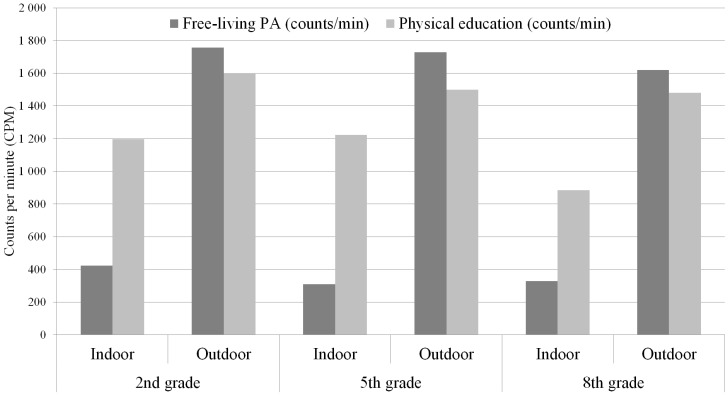
PA intensity levels (CPM) per grade, indoors and outdoors during free-living PA and PE, Sweden 2012–2013; Free living PA indoors differs between grades (*p* < 0.001).

CPM during outdoor free-living PA was 1731.4 (±1005), and yielded 109,742 (±108,031) MVPA counts, whereas CPM during indoor free-living PA was 354.0 (±216.6) and yielded 52,426 (±53,905) MVPA counts (*p* < 0.01). In both girls and boys, free-living CPM outdoors was approximately five-fold that of indoor CPM (Table 3, Figure 2).

This pattern was observed irrespective of grade. PE outdoors yielded higher CPM as well. The contribution of outdoor PE to increase CPM was particularly pronounced among eighth graders (Figure 2).

In terms of free-living PA expressed as CPM, the second graders were significantly more active than fifth and eighth graders (*p* < 0.001)—indoors, but not outdoors (Figure 2).

The second graders accumulated total accelerometer count was 326,366 ± 16,206, *p* < 0.001 vs. fifth (219,422 ± 110,161, *p* < 0.001) and eighth graders (164,783 ± 139,168, *p* < 0.001). Sedentariness generated a mean of 1485.5 (± 463.5) counts per school day, representing 0.7% of total counts.

### 3.4. Physical Activity, Weather, and Season

Mean total daily MVPA outdoor counts were in September 112,679 (±96,615), in March 66,476 (±54,320), and highest in May 148,288 (±138,689) (*p* < 0.01). Indoor MVPA counts were in September 49,104 (±52,033), in March 64,786 (±61,225), and in May 42,823 (±45,742), and thus highest in March (*p* < 0.001). The weather in March and May was mostly sunny compared to more cloudiness and one day with precipitation in one location in September. MVPA counts outdoors declined significantly (*p* < 0.01) as the weather became increasingly inclement (increasing weather index) throughout all grades.

**Figure 3:**
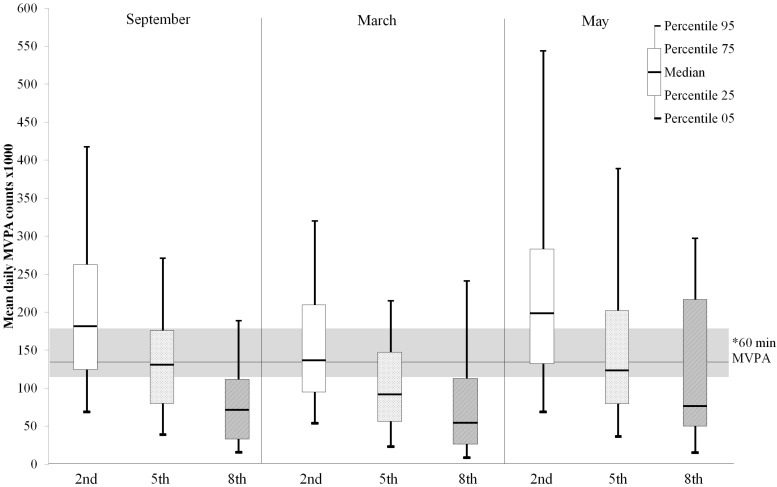
Swedish pupils’ mean daily MVPA counts (×1000) by season and grade; * Children who reached the recommended daily level of 60 min of MVPA during two days or more each season: In September 43 (67.2%) second graders, 39 (43.8%) fifth graders, 1 (3.8%) eighth graders, in March 35 (54.7%) second graders, 19 (21.3%) fifth graders, 8 (30.8%) eighth graders, in May 50 (78.1%) second graders, 41 (46.1%) fifth graders and none of the eighth graders.

Figure 3 illustrates mean total daily counts in MVPA during different seasons in different grades compared to the recommended level of daily 60 min of MVPA (i.e., >137,760 counts in MVPA as by Evensson et al. cut-off point > 2296 CPM) [31].

In terms of mean daily MVPA counts, the second graders achieved recommended levels of MVPA at school during all seasons, which neither the fifth nor eighth graders did. Particularly among eighth graders, mean daily MVPA counts were far below recommended levels of MVPA throughout the seasons (Figure 3).

## 4. Discussion

Free-living PA outdoors was the greatest contributor to daily PA—not only in comparison to indoor stay, but also in comparison to PE in all grades during all seasons, both in terms of mean accelerometer counts and CPM. A commonly-reported environmental impact on school children’s PA is the amount of time spent outdoors [33,34], and our results indicate the significance of outdoor time for increased PA counts during the school day in second, fifth, and eighth graders.

In all grades, there were seasonal differences in total amount of PA counts both indoors and outdoors, and were at their highest during May and September, which could be explained by decreased time outdoors in March. These findings correspond to reported results showing that children are less active in winter than other seasons, generally in areas with cold and long winters [19,35,36]. Thus, winter contains inhibitory factors for PA, at least during school time.

During March the pupils seemed to compensate decreased outdoor activity with increased activity indoors, which is difficult to explain without further behavioral and environmental studies. The strong correlation between time spent outdoors and MVPA has been previously observed [33,37]. Those findings could also explain the higher PA intensities in the second graders, who spent much more time outdoors during the school day than their older peers.

Indoor PE—which one may expect to yield reasonably high levels of CPM—was below CPM levels yielded by free-living PA outdoors, whereas outdoor PE was not infrequently in parity with it. The relatively low volumes of PE counts may be explained by numerous factors. For instance, PE as a subject in the syllabus also contains theoretical elements. Thus, PA may not necessarily be considered as a fundamental part of a PE lesson, at least not in Sweden. In addition, the characteristics of a PE lesson may require youth to engage in instructions and demonstrations, as well as in observations, and this suggests that it is rather challenging to maintain high volumes of MVPA (CPM and counts in MVPA) throughout a PE lesson.

None of the schools participating in our study had scheduled outdoor PE in March, and the question remains why schools do not seize the opportunity to engage the pupils in skiing and skating. Possibly, the arrangements for such lessons are considered too complicated and time-consuming, and the proper facilities may not always be available. Yet, this type of activity would yield good exercise, and moreover, would offer opportunities to combine with outdoor education. Outdoor stay would also, at high latitudes, yield suberythemal sun exposure, which could have a favorable health impact [38]. Prolonged outdoor stay in northern countries during school time deserves to be promoted due to an increase in MVPA in combination with moderate UV exposure, which can lead to positive impacts on the immune system and bone mineralization, and possibly even affect mental health.

Our Swedish participants showed higher levels of MVPA compared to other European studies, with objectively measured PA using the same accelerometer cut-point for MVPA > 2296 CPM [14]. Most of the second graders reached the recommended level of MVPA of 60 min already during school time, which makes the school an important contributor for PA in younger children. However, this did not apply to the older pupils, and among the eighth graders only very few obtained the recommended levels of daily PA. This makes the after school time important in promoting PA in older children and youth. Among eighth graders, PE outdoors contributed to their PA, whereas free-living PA was low, especially in May. During fieldwork in May, eighth graders (the girls in particular) were not infrequently observed to sunbathe, lying down across outdoor benches for the better part of or even entire recess periods, which would not unexpectedly have reduced PA.

Some European studies apply the global positioning system (GPS) for separating outdoor from indoor PA which may be problematic due to common signal inaccuracy and lost outdoor time during the time that it takes for the GPS receiver to find the satellites as the children go outdoors. Separating indoor from outdoor PA requires instruments of high reliability, as for instance the combination of a light sensor and ocular observation, as applied in this study. The strength of this study was thus the use of objective monitoring of outdoor vs. indoor time and the amount and intensity of PA. This is crucial as it is proven challenging to recall details in PA patterns [39]. Further, data for the different seasons were firstly collected within a maximum time span of three weeks for each season, and secondly, for best possible seasonal comparison, the differing latitudes were considered in the order in which the fieldwork was carried out. Thus, during September, fieldwork started in the North, and in March and May in the South. Expensive and time-consuming methods do not seem to be frequently applied in seasonal studies of PA, though important for reliability—which was obvious in this study. During fieldwork, the children were sometimes observed not to adhere to the schedule for lessons and recess which makes direct observation necessary in order to obtain correct data. Finally, using counts in different intensity levels may provide more detailed information about the children’s PA compared to time spent in different intensity levels.

Weaknesses of the study were firstly the limited sample of schools. However, compared to the option of an extensive sample combined with more unreliable fieldwork (e.g., by proxy) our choice—though resource-consuming—was motivated by the variation in school settings and the high accuracy by which data were collected, and the way in- and outdoor stays during the school day have been clocked. Thus, we drew the conclusion that thorough fieldwork in a small sample would deliver more valuable data. Secondly, the drop-out among eighth graders was high, which motivates caution in interpreting these particular data. The calculated power for the eighth graders was still high (>90%). Secondly, the choice of accounting for CPM may have been misguiding in cases of prolonged outdoor stay, particularly among the second graders, as the counts put in relation to the length of outdoor stay may have been diluted. Yet, in absolute terms, the second graders were the most active during free-living PA outdoors.

### Implications for School Health

As a promoter of PA, school health policies should highlight outdoor recess as an important factor for sustained health. As recommended by the WHO, PE should be considered as an integrated part of creating health-promoting environments. In Sweden, the subject of PE includes theory and is not considered to be solely responsible for satisfying the need of school children’s PA. It is possible that this approach needs to be revised. In our investigation, only the second graders obtained the recommended levels of PA as a mean, which none of the others did during any season in terms of mean values. Particularly, the eighth graders’ low levels of PA is a reason for concern. To promote health, the school administrators should provide more opportunities for pupils to get the benefits of outdoor recess during the school day, especially for girls in the upper grades. Outdoor recess is even more important during winter, particularly when there is no scheduled outdoor PE. Additionally, the opportunities for both outdoor PE and theoretical outdoor education during all seasons (winter included) should be explored, as both may have the potential to trigger additional and initially unplanned mobility which does not fall outside the frame of the lesson. Also, the share of the pupils below the 75th percentile of PA need to be considered, and an expansion of outdoor education could serve to push them towards obtaining desirable and recommended levels of PA. In Sweden, in recent years, schools have been established without any access to the surrounding outdoor environment. Even though the implications for PA are discussed in this context, this trend has to be carefully monitored where free-living PA outdoors during school time is concerned, due to its valuable contribution to maintaining high levels of MVPA.

## 5. Conclusions

We conclude that an increase of time spent outdoors—during all seasons—would increase MVPA in children of all ages. Further, free-living outdoor recess is the best contributor to MVPA in lower grades. For the older pupils, outdoor PE is crucial for increased time spent in MVPA during the school day. Factors at school that impact children’s time spent in MVPA outdoors (e.g., theoretical outdoor education) during all seasons should be further explored.

## Figures and Tables

**Table 1:** Mean and standard deviation (SD) of participant’s age and BMI in March, and outdoor time in different grades and seasons.

Grade		Age in March (years)	BMI in March (kg/m^2^)	Outdoor Time in September (min)	Outdoor Time in March (min)	Outdoor Time in May (min)
		Mean (SD)	Mean (SD)	Mean (SD)	Mean (SD)	Mean (SD)
2nd grade	Boys (*n* = 33), Girls (*n* = 31)	8.7 (0.5)	17.3 (1.8)	113.9 (64.1)	85.8.0 (43.6)	128.8 (53.7)
5th grade	Boys (*n* = 43), Girls (*n* = 46)	11.7 (0.3)	19.5 (3.3)	77.5 (41.5)	57.6 (52.1)	77.0 (45.1)
8th grade	Boys (*n* = 16), Girls (*n* = 10)	14.7 (0.3)	21.7 (2.6)	42.3 (36.7)	17.7 (14.5)	60.0 (37.3)

**Table 2:** Predictors for physical activity during school stay (accelerometer counts), non-significant *p*-values not shown.

The GENMOD Procedure, Repeated for Season and Measure Day, Estimate (Standard Error, SE)	Dependent Variable: Total Daily Accelerometer Counts				
Variable	(I)	(J)	Estimate ** (Diff. I-J)	SE	*p*-Value
Season	March (ref.)	May	−44,857	20,823.0	0.0312
Measured day *	Day1 (ref.)	Day 2	14,765	7146.4	0.0388
		Day 3	39,306	10,137.0	0.0001
		Day 4	46,678	8027.0	<0.0001
		Day 5	39,723	8820.0	<0.0001
	Day 2 (ref.)	Day 3	24,541	9730.6	0.0117
		Day 4	31,914	8117.8	<0.0001
		Day 5	24,958	8647.0	0.0039
Grade	2nd grade (ref.)	5th grade	67,497	10,839.0	<0.0001
		8th grade	86,440	15,032.0	<0.0001
Gender	Girl (ref.)	Boy	−32,194	9254.0	0.0005
Weather	Cloudless (ref.)	grey cloudiness	28,328	8511.6	0.0009
		partly cloudy	13,213	5760.0	0.0218
	Grey cloudiness (ref.)	partly cloudy	−15,115	7432.0	0.0420
		white cloudiness	−21,460	8730.4	0.0140
Time outdoors (min)			1076.7	71.8	<0.0001

* 1 = Monday, 2 = Tuesday, 3 = Wednesday, 4 = Thursday, 5 = Friday, and for two schools in May: 1 = Thursday, 2 = Friday, 3 = Monday, 4 = Tuesday, 5 = Wednesday; ** The estimate is the difference between the reference value (I) and the comparative value (J), but for the variable “time outdoors” the estimate means that by each time unit (minute) outdoors the total daily accelerometer counts increase.

**Table 3:** Differences in accelerometer counts-per-minute (CPM) and moderate-to-vigorous PA (MVPA) counts indoors vs. outdoors during free-living PA and PE.

Physical Activity	CPM			MVPA Counts		
	Mean	SD	*p*-Value	Mean	SD	*p*-Value
Free-living PA outdoors	1715.6	522.3	**<0.0001**	91,395.6	59,665.6	**<0.0001**
Free-living PA indoors	358.4	139.4		48,766.1	29,701.7	
Free-living PA outdoors, girls	1698.9	541.9	**<0.0001**	94,443.6	69,621.3	**<0.0001**
Free-living PA indoors, girls	313.1	97.4		39,555.9	20,853.4	
Free-living PA outdoors, boys	1731.3	505.5	**0.0002**	88,513.2	48,635.4	**<0.0001**
Free-living PA indoors, boys	401.3	158.8		57,475.7	34,012.4	
PE outdoors	1494.4	608.4	**<0.0001**	56,496.9	30,555.1	**<0.0001**
PE indoors	1204.1	749.7		41,313.7	32,561.3	
PE outdoors, girls	1333.6	547.0	0.0690	48,637.6	27,136.0	0.0909
PE indoors, girls	1133.8	630.6		38,989.7	29,065.7	
PE outdoors, boys	1649.3	627.2	**<0.0001**	64,072.1	31,885.7	**<0.0001**
PE indoors, boys	1265.0	839.3		43,325.6	35,404.8	

## Abbreviations

The following abbreviations are used in this manuscript:

CPM	Counts per minute
PA	Physical activity
LPA	Light physical activity
MVPA	Moderate to vigorous physical activity
PE	Physical education
SED	Sedentariness
